# Interleukin-35 pathobiology in periodontal disease: a systematic scoping review

**DOI:** 10.1186/s12903-021-01515-1

**Published:** 2021-03-20

**Authors:** Patrick R. Schmidlin, Mandana Dehghannejad, Omid Fakheran

**Affiliations:** 1 Clinic of Conservative and Preventive Dentistry, Center of Dental Medicine, University of Zurich, Plattenstrasse 11, 8032 Zurich, Switzerland; 2 Dental Research Center, Dental Research Institute, Isfahan University of Medical Sciences, Isfahan, Iran; 3 Department of Oral Health and Community Dentistry, Dental Implant Research Center, Dental Research Institute, Isfahan University of Medical Sciences, Isfahan, Iran

**Keywords:** Interleukin-35, Periodontal disease, Systematic review

## Abstract

**Background:**

Interleukin (IL)-35 is a novel anti-inflammatory cytokine that is produced by regulatory T cells. IL-35 mediates immunological functions and plays a protective role in several diseases such as asthma and rheumatoid arthritis. However, the role of IL-35 in gingivitis and periodontitis remains unclear. The aim of this study was to systematically review the literature and collecting the available evidence regarding the role of IL-35 in pathogenesis of periodontal disease.

**Methods:**

A systematic search of electronic databases including MEDLINE, Google Scholar, Cochrane Library, Web of Science, and Scopus was conducted in November 2020 to identify studies addressing the Interleukin-35 pathobiology in periodontal disease. The identified studies were subjected to pre-identified inclusion criteria. The retrived papers were assessed by the authours independently and consensus was reached in cases where disagreement occurred. Articles written in languages other than English, case reports, letters to editors, conference abstracts, theses, and dissertations were excluded from the review.

**Results:**

A total of 176 possibly relevant articles were identified through the search strategy. Finally, 15 papers which met the criteria of eligibility were included in this review by consensus. The included articles were classified based on their design and level of evidence.Three subclinical study, ten cross sectional investigation and two randomized clinical trials constituted the final set of studies in this review. At preclinical level, Il-35 showed inhibitory characteristics regarding alveolar bone resorption of animal periodontitis models. The results of observatory human studies confirmed the presence of high levels of IL-35 in saliva, GCF, serum, and gingival biopsies of patients suffering from inflammatory periodontal disease. Moreover, two included clinical trials showed that non-surgical periodontal therapy could downregulate IL-35 production in chronic periodontitis patients.

**Conclusion:**

Interleukin-35 has an undeniable role in pathobiology of inflammatory periodontal disease. Further well-controlled studies are needed to better elucidate the functional pattern of IL-35 in pathogeneisis of gingival and periodontal disease.

**Supplementary Information:**

The online version contains supplementary material available at 10.1186/s12903-021-01515-1.

## Background

Studies revealed that periodontitis is the sixth most common disease globally. The prevalence of periodontitis is high, and approximately 10% of the global population has been affected by severe periodontitis[1]. As such, it affects the tooth supporting tissues leading to attachment loss and—if left untreated—to tooth loss. It represents a multifactorial disease primarily caused by pathogenic bacteria, which triggers and maintains a host-mediated process [2, 3]. The interplay between the imbalance within the microbial community and a dysregulated host immune response—known as dysbiosis—including some systemic and environmental modifying risk-factors leads to destruction of periodontal tissues, i.e., the periodontal ligament and alveolar bone [4]. Several studies have documented that cell-produced cytokines play a critical role in this process [5, 6], as they up- and down- regulated the local cell environment.

Different types of cells, such as fibroblasts, macrophages, and lymphocytes secrete a plethora of cytokines, especially during the active phases of periodontitis. Among the first cytokines discovered within the context of periodontal inflammation were the interleukin-1 (IL-1), IL-6, and tumor necrosis factor (TNF)families [7].

These cytokines are primarily recognized as pro-inflammatory cytokines exhibiting mainly pleiotropic effects on lymphocyte promotion and tissue destruction [8]. Further studies have documented some important related cytokines such as IL-1 receptor antagonist (IL-1Ra), IL-10, IL-4, IL-11, and transforming growth factor-beta [TGFB] with anti-inflammatory functions within the destructive processes [9]. It has been shown that both pro-inflammatory and anti-inflammatory cytokines play a critical role in the pathogenesis of periodontitis, thereby influencing destruction, repair, and remodeling of periodontal tissues [10, 11]. In this context, IL-35 is a rather newly discovered cytokine with shown anti-inflammatory capacities [12]. It is mainly produced by regulatory T cells (Treg) and formed from a heterodimer of IL-12p35 subunits and Epstein-Barr virus induced gene 3 (EBI3) [13]. The binding of IL-35 and its receptors, IL-12Rβ2, gp130, or a heterodimer of IL-12Rβ2:gp130 activates the Janus kinase–signal transducer and activator of transcription (JAK-STAT) pathway and induces immunosuppression[14].In this regard, in a gene polymorphism analyses study among Japanese subjects, the authors reported that the frequencies of variant alleles of IL-12Rβ2 were significantly higher in aggressive periodontitis patients as compared with healthy controls or chronic periodontitis patients.

It has been documented that IL-35 mainly suppresses proliferation of T cells by blocking mitosis in G1 phase without provoking apoptosis [15, 16]. IT can also induce the development of IL-35-producing T cells (iTr35), which are subsets of regulatory T cells (Treg) [16] and inhibits IL-17 production by inhibiting T-helper-17 (Th17) cell induction and, thus, plays a protective role in several diseases, whose pathogenesis is closely associated with the Th17/Treg imbalance, such as experimental colitis, asthma, inflammatory bowel disease, and rheumatoid arthritis [17–20].

IL-35 is also secreted by B cells and induces the conversion of human B cells into Breg cells to inhibit antimicrobial immunity through production of IL-35. This cytokine may also play a crucial role in the immune suppression function of Breg cells[21, 22].

Moreover, a recently published experimental study’s findings revealed that IL-35 prevents bone loss in rheumatoid arthritis disease [23]. IL-35 has also been detected in periodontal tissues, gingival crevicular fluid (GCF) of different types of periodontal disease [24–26] and some evidence supports its important role in the pathogenesis of periodontitis [27, 28]. The iTr35 cells also negatively regulate various immune responses mediated by Th1, Th17, and cytotoxic T lymphocyte cells. It has been documented that all of these immune cells are involved in pathogenesis of periodontitis [12, 29–32].

Previously it has been documented that through the production of IL-10, IL-35, and transforming growth factor b (TGF-b), Breg cells suppress immunopathology by prohibiting the expansion of pathogenic T cells and other pro-inflammatory lymphocytes[33]. Recently the results of a human study approved that the frequency of Breg cells was positively correlated to serum levels of IL10, IL35, and TGFβ1 in peripheral blood samples of periodontitis patients[34].

Periodontitis may be viewed as an infectious disease with a number of specific characteristics[35]. Pathogens of the subgingival microbiota can interact with host tissues and activate related immune responses. Recent studies suggested that IL-35 may promote infectious tolerance in some ways [21, 36–38]. In this regard the results of an animal study showed that the mice which did not express IL-35 displayed a strikingly improved resistance to infection with the intracellular bacterial pathogen *Salmonella typhimurium*[37]. These experimental samples showed superior containment of the bacterial growth and prolonged survival both after primary infection and upon secondary challenge after vaccination, compared to control group. This ability of Il-35 should be also considered in the way of describing the role of IL-35 in pathogenesis of periodontal disease.

To the best of our knowledge, there is no systematically collected evidence in the literature in this regard, especially in the field of periodontology. Hence, this study aimed to systematically review the existing literature regarding the role of IL-35 in pathogenesis of periodontal disease.

## Methods

### Scoping review approach and related question

A literature review using a scoping review approach, which is commonly used to elaborate and better understand the available evidence on a given topic, identify gaps in the literature and assess the need for further research [39]. Arksey and O’Malley explained the required methodological framework stages for a scoping review [39] and served as the methodological basis for this work. The main modified focus question of this study was as follows: ‘’What is known from the available literature about the role of IL-35 in the pathogenesis and controlling of periodontal disease?’’.

### Search strategy

Our literature search applied a wide range of computerized databases, including MEDLINE, Google Scholar, Cochrane Library, Web of Science, and Scopus. The following search terms included relevant keywords in various forms and combinations are used in the study: Periodontitis, gingivitis, and interleukin-35 and interleukin-35 receptors (IL-12Rβ2, gp130). Full details of the search strings used in this review are shown in additional file 1. It should be noted that the search syntax was adapted to each database based on their specific instructions.

### Eligibility criteria

**Fig. 1:**
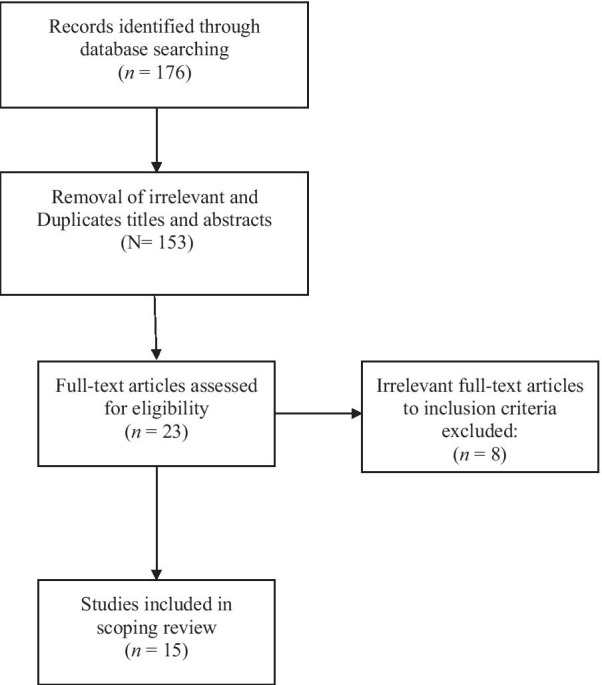
Flowchart of the process for study selection

Retrieved titles and abstracts obtained from the search, along with the full texts (if necessary), were assessed by two independent reviewers (OF and MD). Any inconsistencies in this regard were solved by a constructive discussion. After completing this initial search, the reference lists of the selected articles were also reviewed to identify additional relevant studies not found during the original electronic search. All letters, narrative reviews, and duplicate articles were excluded. The search strategy was not restricted by the publication date. Hence, all relevant evidence that met the inclusion criteria by November 20, 2020, was assessed. The process of selection articles for the final review is illustrated in Fig. 1.

### Screening of studies and data extraction

Two authors (OF and MD) searched the databases independently. The two sets of articles were then compared. Disagreements were resolved through discussion or, if necessary, by including a third researcher (PS) to make the final decision. Duplicate articles were also excluded from the review. One researcher (OF) extracted the data, and another author (PS) checked its accuracy and completeness. The final set of selected articles and the relevant data based on the study research question is summarized in Table 1.

**Table 1:** General characteristics and main outcomes of the included studies

Article	Design	Results	Authors’ conclusion
Kamiya 2020 [42]	Invitro Study	In this study, IL-35 and RANKL induced osteoclastogenesis synergistically	Osteoclastogenesis activity
Shindo 2019 [41]	Invitro study	Results of this study indicate that IL-35 plays a role in regulating the progression of periodontitis by inhibiting inflammatory cytokine (IL-6 and IL-8) production	Anti-inflammatory activity
Cafferata 2020 [43]	Animal Study (locally & systemically administrated IL-35)	IL-35 inhibited alveolar bone resorption in periodontitis mice IL-35 inhibited alveolar bone resorption and this inhibition was closely associated with modulation of the periodontal Th17/Treg imbalance	Anti-inflammatory activity
Cai 2017 [45]	Cross- sectional (GCF and Serum samples)	The study confirmed the presence of high levels of IL-35 on GCF (or blood) of CP patients. Moreover, high concentrations of IL-35 on GCF may relate to the levels of them on blood Elevated IL-35-related sCD14 is associated with the severity of periodontitis	Pivotal role in pathogenesis of periodontitis
Costantini 2020 [61]	Cross- sectional (Saliva Samples)	An increase in IL-35 salivary level was observed in periodontopathic patients with respect to the healthy controls	Pivotal role in pathogenesis of periodontitis
Hetta 2020 [34]	Cross- sectional (Serum Samples)	Serum levels of IL-35 were significantly higher in patients with periodontitis compared to the healthy controls	Pivotal role in pathogenesis of periodontitis
Jin 2017 [27]	Cross-sectional (GCF, Serum, periodontal tissues Samples)	IL-35 protein and periodontal clinical indicators were negatively correlated. It was hypothesized that the increased level of IL-35 plays a protective role in periodontal disease by maintaining immune system homeostasis and dampening the inflammatory response, and highlights IL-35 as a potential new therapy for the treatment of periodontitis	Anti-inflammatory activity
Jing 2019 [28]	Cross-sectional (gingival biopsies)	This study identified a subset of plasma cell which produces IL-35 and IL-37. This may regulate periodontitis pathogenesis by inhibiting alveolar bone loss through directly blocking osteoclast formation	Anti-inflammatory activity
Kalburgi 2013 [26]	Cross-sectional (gingival biopsies)	The increased expression of IL-35 in chronic and aggressive periodontitis suggests its possible role in pathogenesis of periodontitis	Pivotal role in pathogenesis of periodontitis
Köseoğlu 2015 [24]	Cross-sectional (GCF, Serum and Plasma samples)	IL-35 could have an important role to suppress the periodontal inflammation and maintain periodontal health	Anti-inflammatory activity
Maboudi 2019 [44]	Cross- sectional (GCF and Serum samples)	neither Type 2 diabetes mellitus nor chronic periodontitis differentially affects serum levels of IL-35	No significant association
Mitani 2015 [25]	Cross-sectional (GCF samples and gingival biopsies)	IL-35 level was significantly higher in GCF and gingival tissues of patients with periodontitis than healthy participants	Pivotal role in pathogenesis of periodontitis
Okada 2017 [62]	Cross-sectional (Peripheral blood mononuclear cells)	IL-35 could directly suppress IL-17 expression via RORα and RORγ t inhibition to restrain the excessive immune response in inflammatory conditions such as periodontitis	Anti-inflammatory activity
Raj 2018 [47]	Clinical trial (nonsurgical periodontal treatment) (GCF and Serum Samples)	GCF and serum IL‑35 concentration among Chronic periodontitis individuals was highest among all the groups. Individuals receiving NSPT showed a significant reduction in IL‑35 levels as compared to CP individuals	Pivotal role in pathogenesis of periodontitis
Üstün 2018 [48]	Clinical trial (GCF samples)	The level of IL-35 significantly reduced after SRP in chronic periodontitis patients	Pivotal role in pathogenesis of periodontitis

### Exclusion criteria

Articles written in languages other than English, studies focusing on medically compromised individuals, case reports/case series, letters to editors, opinions, conference abstracts, commentaries, theses, and dissertations were excluded from the review.

#### Results

### Study selection

A total of 176 possibly relevant articles were identified through the search strategy. After completing the screening titles and abstracts and eliminating duplicates, 23 studies were retrieved, and their full-text versions were collected for further assessment. A manual search for the reference lists of the 23 studies revealed no additional qualifying paper. Out of these 23 studies were subjected to full article review eligibility, and eight articles were excluded. The reasons for excluding full-text articles are as follows: The English versions of the full-text articles were not available (n = 3), the articles were not suitable for our research question (n = 2), and articles consisting of literature reviews without any original data (n = 3). Finally, 15 articles were found to be eligible and could be included in this review. Additional details on the data search are shown in the flowchart (Fig. 1).

### Results of included studies

We classified and presented the results of fifteen articles included in this review based on their design and level of evidence[40]. In the first subchapter, we presented the results of two in vitro cell studies and one animal (preclinical) study. In the second subchapter, including the most articles, we described the results of ten cross-sectional studies. Finally, in the last subchapter, the results and outcomes of two human randomized control trials (RCT) regarding the effect of non-surgical periodontal treatment on levels of IL-35 were presented. The detailed characteristics of included studies are summarized in Table 1.

### *Animal and *In vitro* cell studies*

In the first in vitro cell study by Shindo et al., who used human periodontal ligament cells stimulated with IL-17A [41]. In this research, the authors reported that IL-35 produced from regulatory T cells might inhibit progression of periodontitis by decreasing IL-17A-induced levels of IL-6 and IL-8. Another in vitro cell study, using cultured mouse monocyte line RAW cells, osteoclastogenesis function of IL-35 was assessed [42]. They stimulated RANKL-treated RAW cells with or without IL-35 to determine whether IL-35 affects RANKL-dependent osteoclastogenesis. The results showed that IL-35 and RANKL induced osteoclastogenesis synergistically. In the last study in this category, an interventional animal study was conducted on mice with ligature-induced periodontitis[43]. In this study, the researchers evaluated the effect of locally or systemically administered IL-35 on alveolar bone resorption measured by micro-computed tomography and scanning electron microscopy. The results of this project revealed that IL-35 inhibited alveolar bone resorption in periodontitis mice. Furthermore, IL-35 induced less detection of Th17 lymphocytes and down regulation of Th17-related cytokines, along with higher detection of Treg lymphocytes and upregulation of Treg-related cytokines in periodontitis-affected tissues. The authors of this article also reported that in periodontitis-affected mice treated with IL-35, the mRNA and protein levels of RANKL were significantly lower and conversely the expressed and secreted levels of OPG were significantly higher comparing to mice without IL_35 treatment.

### Association studies with cross-sectional design

Ten of the studies included in this review were mainly association studies with a cross-sectional design. Almost all of these studies compared the level of IL-35 in various tissue samples, such as peripheral blood, serum, gingival crevicular fluid (GCF), and periodontal tissue biopsies in periodontitis patients with a healthy control group. Among these, nine studies were conducted among systemically healthy individuals, and only one study was conducted among patients with Type 2 diabetes mellitus. The study results among patients with diabetes showed that neither type 2 diabetes mellitus nor chronic periodontitis differentially has affected serum levels of IL-35 [44]. However, the results of all the studies among systematically healthy individuals showed that the levels of IL-35 were higher in tissue samples of periodontitis patients comparing to non-infected samples. In this regard, eight studies focused on chronic types of periodontal disease, including gingivitis and periodontitis. However, Kalburgi et al. examined IL-35 mRNA expression in gingival tissue samples of patients with aggressive periodontitis, patients with chronic periodontitis, and healthy controls using reverse transcriptase polymerase chain reaction (RT-PCR). The results showed that IL-35 mRNA expression was highest in subjects with chronic periodontitis compared to the subjects with aggressive periodontitis and the least seen in healthy patients[26]. Among the included studies, only one paper reported that IL-35 elevated level was also associated with the severity of chronic periodontitis[45]. Unfortunately, due to substantial heterogeneity in sampling methods, severity of disease, and laboratory tests used in these observational studies, the post hoc analysis such as meta-analysis was not feasible [46].

### Randomized control clinical studies

Only two human interventional studies on IL-35 (patho) biology in periodontal disease were identified [47, 48]. Both were randomized controlled clinical trials and assessed the effect of non-surgical periodontal treatment (NSPT) on IL-35 level among patients with chronic periodontitis. The first study was conducted among forty patients with chronic periodontitis who were randomly divided into two equal groups [48]. The control group received scaling root planning (SRP), and the test group treated with SRP followed by Er, Cr: YSGG laser. The results of this trial showed that all clinical parameters and GCF levels of immunological factors (IL-1β, IL-6, IL-35) significantly reduced in both groups compared with the baseline (P < 0.05), but no significant change was detected among the groups.

In the second study, 60 participants were divided into three equal groups, including healthy, gingivitis, and chronic periodontitis patients [47]. Only the subjects in periodontitis group underwent SRP treatment completed in two sessions within 24 h in accordance with the Quirynen’s one stage, full mouth debridement protocol. The study findings revealed that the mean IL-35 concentration in GCF samples of patients with chronic periodontitis was significantly higher than the other groups at baseline. Furthermore, the results confirmed a significant reduction in IL-35 concentration of GCF samples obtained from patients with periodontal disease six weeks after NSPT. It should be noted that this reduction of IL-35 concentration was consistent with the decrease in clinical parameters, including periodontal pocket depth (PPD), Clinical attachment level (CAL), plaque index (PI), gingival index (GI) and bleeding index (BI) in patients with periodontal disease. As there is a high level of heterogeneity in periodontal disease definitions, treatment protocols, and laboratory tests used in these studies, it was not appropriate to apply meta-analysis.

#### Discussion

IL-35 has been introduced as an anti-inflammatory cytokine in various diseases and conditions, such as experimental colitis, allergic asthma, and collagen-induced autoimmune arthritis [15, 17, 49]. IL-35 is—relatively spoken—a newcomer among suppressive cytokines[12]. The results of many studies showed the role of Th1 and Th17 cells and their cytokine profiles in periodontal disease [31, 50–53]. Based on the results of various studies, abnormal cellular immunity occurs in chronic periodontitis and elevated peripheral Th17 and Th1 cells might be simultaneously associated with the development of periodontal disease[31, 50]. It is interesting that IL-35 inhibits Th17 and Th1 cells and may play a protective role in periodontal disease[22, 54].

In this study, we tried to collect all available data in the literature about the role of IL-35 in pathobiology of periodontal disease. An in-depth analysis of the role of IL-35 could provide novel avenues for diagnosing, monitoring, and treating periodontitis.

In vitro and preclinical animal studies provided critical information about the function of IL-35 and the interaction of other immunological factors with this newly found agent. A study by Shindo et al. suggested an anti-inhibitory action for IL-35 in pathogenesis of periodontitis [41] and reported that IL-35 could inhibit the production of inflammatory cytokines such as IL-6 and IL-8. In this line, the results of an interventional animal study also considered an inhibitory effect for IL-35 in pathogenesis of alveolar bone resorption [43]. Another in vitro cell study suggested that IL-35 may also induce osteoclastogenesis by a synergistic correlation with RANKL [42]. It should be noted that in this study, the researchers just evaluated one type of cells (RAW264). However as they declared in their report, bone marrow derived-macrophages include many cell populations, mainly lymphocytes, which may affected by IL-35 in various manners [42]. In this regard, the results from the cross-sectional and RCTs were more consistent. All of these studies, except one conducted on patients with diabetes, confirmed the higher levels of IL-35 in tissue samples of patients with periodontal disease.

In only one study evaluating serum levels of IL-35 in patients with periodontal disease, included type 2 diabetic individuals with a control group, the results were inconsistent with the others[44]. The authors of this study reported that, despite significant associations of serum concentration of IL-35 with certain periodontal and inflammatory indices, neither type 2 diabetes mellitus nor chronic periodontitis affected the serum levels of these cytokines differently. This contradictory result may be related to the evaluation of this biomarker in the serum, not with GCF or a gingival biopsy.

In this way, the results of two interventional randomized clinical trials proved that treatment of chronic periodontitis and controlling periodontal tissues' inflammation could reduce the concentration of IL-35 in GCF. The exact function of IL-35 in etiopathogenesis of periodontal disease is not clear, but it is suggested to be produced by Treg cells as negative feedback regulation[12]. Previous research has revealed that in chronic periodontitis, the expression of Treg cells is significantly increased compared to gingivitis and healthy condition [55, 56]. These cells are recruited by immune system for arresting tissue destruction and containing the disease. Hence, a reduction in inflammatory load reduces the expression of Treg cells. Therefore, IL-35 levels in GCF also decrease. This may explain the significant reduction in GCF IL-35 levels in chronic patients with periodontal disease after NSPT was performed in two included RCTs [47, 48].

The results of the included studies in this review do not contradict previous reports on the protective role of IL-35 in pathogenesis of inflammatory diseases. Loss of IL-35 activity in animal models leads to worsening and progression of various inflammatory diseases such as encephalomyelitis [57, 58]. In this regard, samples lacking functional IL-35 showed greater severity of the disease[59, 60]. Conversely, when IL-35 was administered as a therapeutic agent in collagen-induced rheumatoid arthritis-affected mice, a significant reduction of pathological inflammation was observed[20]. In this scope, there is only one study in periodontology conducted by Cafferata et al. examining the effect of IL-35 administration on the alveolar bone resorption in periodontitis mice [43]. They concluded that IL-35 is beneficial in the regulation of periodontitis. Particularly, IL-35 inhibited alveolar bone resorption, and this inhibition was closely associated with modulation of the periodontal Th17/Treg imbalance.

Most of the studies included in this review had a cross-sectional design (level III) and thus indicated an overall low level of evidence to prove the definitive role of IL-35 in periodontitis[40]. Further longitudinal and controlled clinical trials are required to clarify the detailed functional pattern of IL-35 in various periodontal diseases. Future research in the IL-35 field could develop new innovative treatment methods to control periodontal.

#### Conclusion

In summary, these scoping review results confirmed the presence of high levels of IL-35 in saliva, GCF, serum, and gingival biopsies of patients with clinically diagnosed inflammatory periodontal disease. Elevated levels of IL-35 in periodontal tissues might be associated with the severity of periodontitis. At preclinical level, IL-35 showed inhibitory characteristics regarding alveolar bone resorption of periodontitis models. Moreover, non-surgical periodontal therapy could downregulate IL-35 production in patients with chronic periodontal disease.

## Supplementary Information

**Additional file 1.** Full details of the search protocol used in this systematic review.

## Data Availability

The datasets supporting the conclusions of this article are included within the article and its additional file.

## Abbreviations

IL: Interleukin
TNF: Tumor necrosis factor
IL-1Ra: Interleukin 1 receptor antagonist
TGFB: Transforming growth factor-beta
Treg: Regulatory T cells
EBI3: Epstein-Barr virus induced gene 3
Th17: T-helper-17
RCT: Randomized control trials
GCF: Gingival crevicular fluid
RT-PCR: Reverse transcriptase polymerase chain reaction
NSPT: Non-surgical periodontal treatment
SRP: Scaling root planning
PPD: Periodontal pocket depth
CAL: Clinical attachment level
PI: Plaque index
GI: Gingival index
BI: Bleeding index
