# Supplementary material for: Interleukin-35 pathobiology in periodontal disease: a systematic scoping review
Source: BMC Oral Health. 2021 Mar 20;21:139. doi: 10.1186/s12903-021-01515-1 (PMC7981974; doi:10.1186/s12903-021-01515-1)
Supplement: Supplementary file 1 — Additional file 1. Full details of the search protocol used in this systematic review. [file 12903_2021_1515_MOESM1_ESM.docx]

Supplementary 1: Full details of the search protocol used in this systematic review.

November 20, 2020

(((periodont*[Title/Abstract]) OR gingiv*[Title/Abstract])) AND ((((il-35[Title/Abstract]) OR il 35[Title/Abstract]) OR interleukin-35[Title/Abstract]) OR interleukin 35[Title/Abstract])

Search details:

((periodont[Title/Abstract] OR periodont's[Title/Abstract] OR periodonta[Title/Abstract] OR periodontain[Title/Abstract] OR periodontais[Title/Abstract] OR periodontal[Title/Abstract] OR periodontal'[Title/Abstract] OR periodontalfibroblast[Title/Abstract] OR periodontalligament[Title/Abstract] OR periodontally[Title/Abstract] OR periodontallyaccelerated[Title/Abstract] OR periodontalogy[Title/Abstract] OR periodontalpathogens[Title/Abstract] OR periodontalprobe[Title/Abstract] OR periodontalspecific[Title/Abstract] OR periodontaltitis[Title/Abstract] OR periodontaly[Title/Abstract] OR periodontapathies[Title/Abstract] OR periodonte[Title/Abstract] OR periodontemetry[Title/Abstract] OR periodontia[Title/Abstract] OR periodontial[Title/Abstract] OR periodontially[Title/Abstract] OR periodontic[Title/Abstract] OR periodontical[Title/Abstract] OR periodontically[Title/Abstract] OR periodontics[Title/Abstract] OR periodontics'[Title/Abstract] OR periodonticum[Title/Abstract] OR periodontii[Title/Abstract] OR periodontilysis[Title/Abstract] OR periodontinum[Title/Abstract] OR periodontio[Title/Abstract] OR periodontis[Title/Abstract] OR periodontisis[Title/Abstract] OR periodontist[Title/Abstract] OR periodontist'[Title/Abstract] OR periodontist's[Title/Abstract] OR periodontistis[Title/Abstract] OR periodontists[Title/Abstract] OR periodontists'[Title/Abstract] OR periodontit[Title/Abstract] OR periodontital[Title/Abstract] OR periodontite[Title/Abstract] OR periodontites[Title/Abstract] OR periodontitic[Title/Abstract] OR periodontitides[Title/Abstract] OR periodontities[Title/Abstract] OR periodontitis[Title/Abstract] OR periodontitis'[Title/Abstract] OR periodontitis's[Title/Abstract] OR periodontitisassociated[Title/Abstract] OR periodontitisinduced[Title/Abstract] OR periodontitisis[Title/Abstract] OR periodontitislesions[Title/Abstract] OR periodontitispatient[Title/Abstract] OR periodontitispatients[Title/Abstract] OR periodontititis[Title/Abstract] OR periodontitits[Title/Abstract] OR periodontitium[Title/Abstract] OR periodontits[Title/Abstract] OR periodontitsts[Title/Abstract] OR periodontitus[Title/Abstract] OR periodontium[Title/Abstract] OR periodontium'[Title/Abstract] OR periodontium's[Title/Abstract] OR periodontiumare[Title/Abstract] OR periodontiumiatrogenically[Title/Abstract] OR periodontiums[Title/Abstract] OR periodontiun[Title/Abstract] OR periodontl[Title/Abstract] OR periodonto[Title/Abstract] OR periodontoal[Title/Abstract] OR periodontoblast[Title/Abstract] OR periodontoblastic[Title/Abstract] OR periodontoclasia[Title/Abstract] OR periodontogastric[Title/Abstract] OR periodontogenesis[Title/Abstract] OR periodontogenic[Title/Abstract] OR periodontogram[Title/Abstract] OR periodontograms[Title/Abstract] OR periodontography[Title/Abstract] OR periodontoic[Title/Abstract] OR periodontoid[Title/Abstract] OR periodontoidal[Title/Abstract] OR periodontoideal[Title/Abstract] OR periodontol[Title/Abstract] OR periodontol2000[Title/Abstract] OR periodontolgy[Title/Abstract] OR periodontolhttp[Title/Abstract] OR periodontologial[Title/Abstract] OR periodontologic[Title/Abstract] OR periodontological[Title/Abstract] OR periodontologically[Title/Abstract] OR periodontologist[Title/Abstract] OR periodontologists[Title/Abstract] OR periodontologists'[Title/Abstract] OR periodontology[Title/Abstract] OR periodontology'[Title/Abstract] OR periodontology'81[Title/Abstract] OR periodontology's[Title/Abstract] OR periodontologyfrom[Title/Abstract] OR periodontoly[Title/Abstract] OR periodontolyses[Title/Abstract] OR periodontolysis[Title/Abstract] OR periodontoma[Title/Abstract] OR periodontomas[Title/Abstract] OR periodontometer[Title/Abstract] OR periodontometric[Title/Abstract] OR periodontometry[Title/Abstract] OR periodontonlogy[Title/Abstract] OR periodontontal[Title/Abstract] OR periodontoology[Title/Abstract] OR periodontopahogenic[Title/Abstract] OR periodontopathia[Title/Abstract] OR periodontopathic[Title/Abstract] OR periodontopathic'[Title/Abstract] OR periodontopathics[Title/Abstract] OR periodontopathies[Title/Abstract] OR periodontopathis[Title/Abstract] OR periodontopatho[Title/Abstract] OR periodontopathogen[Title/Abstract] OR periodontopathogen'[Title/Abstract] OR periodontopathogenc[Title/Abstract] OR periodontopathogenes[Title/Abstract] OR periodontopathogenesis[Title/Abstract] OR periodontopathogenetic[Title/Abstract] OR periodontopathogenic[Title/Abstract] OR periodontopathogenicity[Title/Abstract] OR periodontopathogenics[Title/Abstract] OR periodontopathogenous[Title/Abstract] OR periodontopathogens[Title/Abstract] OR periodontopathogens'[Title/Abstract] OR periodontopathogensis[Title/Abstract] OR periodontopathology[Title/Abstract] OR periodontopathy[Title/Abstract] OR periodontopatic[Title/Abstract] OR periodontopaties[Title/Abstract] OR periodontopatogenic[Title/Abstract] OR periodontophathic[Title/Abstract] OR periodontophatic[Title/Abstract] OR periodontophthy[Title/Abstract] OR periodontoprosthetic[Title/Abstract] OR periodontorheogram[Title/Abstract] OR periodontoses[Title/Abstract] OR periodontosis[Title/Abstract] OR periodontosis'[Title/Abstract] OR periodontotic[Title/Abstract] OR periodontotitis[Title/Abstract] OR periodontotopathogens[Title/Abstract] OR periodontotrauma[Title/Abstract] OR periodontpathic[Title/Abstract] OR periodonts[Title/Abstract] OR periodonttal[Title/Abstract] OR periodonttis[Title/Abstract] OR periodonttitis[Title/Abstract] OR periodontum[Title/Abstract] OR periodonty[Title/Abstract] OR periodontyal[Title/Abstract]) OR (gingiva[Title/Abstract] OR gingiva'[Title/Abstract] OR gingiva's[Title/Abstract] OR gingivaderived[Title/Abstract] OR gingivae[Title/Abstract] OR gingivahyperplasia[Title/Abstract] OR gingivai[Title/Abstract] OR gingivain[Title/Abstract] OR gingivain's[Title/Abstract] OR gingivains[Title/Abstract] OR gingivais[Title/Abstract] OR gingival[Title/Abstract] OR gingival'[Title/Abstract] OR gingivalat[Title/Abstract] OR gingivalderived[Title/Abstract] OR gingivale[Title/Abstract] OR gingivalenlargement[Title/Abstract] OR gingivales[Title/Abstract] OR gingivalgenal[Title/Abstract] OR gingivali[Title/Abstract] OR gingivalils[Title/Abstract] OR gingivalindex[Title/Abstract] OR gingivalis[Title/Abstract] OR gingivalis'[Title/Abstract] OR gingivalis's[Title/Abstract] OR gingivalisand[Title/Abstract] OR gingivalisc[Title/Abstract] OR gingivalisin[Title/Abstract] OR gingivalisinfected[Title/Abstract] OR gingivalisinfection[Title/Abstract] OR gingivalisis[Title/Abstract] OR gingivalislps[Title/Abstract] OR gingivalismp4[Title/Abstract] OR gingivalitis[Title/Abstract] OR gingivallis[Title/Abstract] OR gingivalls[Title/Abstract] OR gingivally[Title/Abstract] OR gingivals[Title/Abstract] OR gingivamol[Title/Abstract] OR gingivamoll[Title/Abstract] OR gingivas[Title/Abstract] OR gingive[Title/Abstract] OR gingive's[Title/Abstract] OR gingivectomies[Title/Abstract] OR gingivectomized[Title/Abstract] OR gingivectomy[Title/Abstract] OR gingivectory[Title/Abstract] OR gingives[Title/Abstract] OR gingivia[Title/Abstract] OR gingivial[Title/Abstract] OR gingivialis[Title/Abstract] OR gingivicanis[Title/Abstract] OR gingivicola[Title/Abstract] OR gingivin[Title/Abstract] OR gingivinus[Title/Abstract] OR gingivite[Title/Abstract] OR gingivitic[Title/Abstract] OR gingivitides[Title/Abstract] OR gingivities[Title/Abstract] OR gingivitis[Title/Abstract] OR gingivitis'[Title/Abstract] OR gingivitises[Title/Abstract] OR gingivitisoral[Title/Abstract] OR gingivititis[Title/Abstract] OR gingivitits[Title/Abstract] OR gingivitol[Title/Abstract] OR gingivits[Title/Abstract] OR gingivl[Title/Abstract] OR gingivlais[Title/Abstract] OR gingivlias[Title/Abstract] OR gingivo[Title/Abstract] OR gingivoalveolar[Title/Abstract] OR gingivoalveoloplasties[Title/Abstract] OR gingivoalveoloplasty[Title/Abstract] OR gingivoaxial[Title/Abstract] OR gingivobucalis[Title/Abstract] OR gingivobuccal[Title/Abstract] OR gingivocclusal[Title/Abstract] OR gingivocervical[Title/Abstract] OR gingivocrevicular[Title/Abstract] OR gingivodental[Title/Abstract] OR gingivodentary[Title/Abstract] OR gingivoestomatitis[Title/Abstract] OR gingivofacial[Title/Abstract] OR gingivofibromatosis[Title/Abstract] OR gingivolabial[Title/Abstract] OR gingivolingual[Title/Abstract] OR gingivolis[Title/Abstract] OR gingivolstomatitis[Title/Abstract] OR gingivoma[Title/Abstract] OR gingivomandibular[Title/Abstract] OR gingivomatosis[Title/Abstract] OR gingivomegaly[Title/Abstract] OR gingivomorphometry[Title/Abstract] OR gingivomucoperiosteal[Title/Abstract] OR gingivomucosa[Title/Abstract] OR gingivomucosal[Title/Abstract] OR gingivomuscosal[Title/Abstract] OR gingivoocclusal[Title/Abstract] OR gingivoocclusally[Title/Abstract] OR gingivopalatal[Title/Abstract] OR gingivopapillary[Title/Abstract] OR gingivopathies[Title/Abstract] OR gingivopathogenic[Title/Abstract] OR gingivopathy[Title/Abstract] OR gingivoperiodontal[Title/Abstract] OR gingivoperioplasty[Title/Abstract] OR gingivoperiosteal[Title/Abstract] OR gingivoperiosteoplasties[Title/Abstract] OR gingivoperiosteoplasty[Title/Abstract] OR gingivoperiostetoplasty[Title/Abstract] OR gingivoperiosteum[Title/Abstract] OR gingivoperiostioplasty[Title/Abstract] OR gingivoperiostoplastie[Title/Abstract] OR gingivoperiostoplasty[Title/Abstract] OR gingivopestibuloplasty[Title/Abstract] OR gingivoplastic[Title/Abstract] OR gingivoplasties[Title/Abstract] OR gingivoplasty[Title/Abstract] OR gingivoproximal[Title/Abstract] OR gingivoproximally[Title/Abstract] OR gingivorhage[Title/Abstract] OR gingivorragia[Title/Abstract] OR gingivorrhagia[Title/Abstract] OR gingivorrhagias[Title/Abstract] OR gingivorrhoea[Title/Abstract] OR gingivosis[Title/Abstract] OR gingivostamtitis[Title/Abstract] OR gingivostomatis[Title/Abstract] OR gingivostomatisis[Title/Abstract] OR gingivostomatitis[Title/Abstract] OR gingivostomatosis[Title/Abstract] OR gingivostomstomatitis[Title/Abstract] OR gingivovestibular[Title/Abstract] OR gingivovestibuloplasty[Title/Abstract] OR gingivox[Title/Abstract] OR gingivox'[Title/Abstract] OR gingivtis[Title/Abstract] OR gingivulis[Title/Abstract])) AND (((il-35[Title/Abstract] OR il 35[Title/Abstract]) OR interleukin-35[Title/Abstract]) OR interleukin 35[Title/Abstract])
